# Bioactive Vitamin D Attenuates MED28-Mediated Cell Growth and Epithelial–Mesenchymal Transition in Human Colorectal Cancer Cells

**DOI:** 10.1155/2022/2268818

**Published:** 2022-08-29

**Authors:** Chun-Yin Huang, Yu-Ting Weng, Nien-Tsu Hsieh, Po-Chen Li, Tzu-Yi Lee, Chun-I Li, Hsiao-Sheng Liu, Ming-Fen Lee

**Affiliations:** ^1^Department of Nutrition, China Medical University, Taichung, Taiwan; ^2^Department of Nutrition and Health Sciences, Chang Jung Christian University, Tainan, Taiwan; ^3^Department of Microbiology and Immunology, National Cheng Kung University, Tainan, Taiwan; ^4^Center for Cancer Research, College of Medicine, Kaohsiung Medical University, Kaohsiung, Taiwan

## Abstract

Inadequate vitamin D status may increase the risk of developing multiple types of cancer. Epidemiological studies suggest an inverse association between 25-hydroxyvitamin D_3_ (25(OH)D_3_) and malignancy, including colorectal cancer. Previous studies have suggested that MED28, a Mediator subunit involved in transcriptional regulation, is associated with the growth of colorectal cancer cells; however, its role in the progression of metastasis such as epithelial–mesenchymal transition (EMT) and cell migration of colorectal cancer is unclear at present. The aim of this study was to investigate a potentially suppressive effect of calcitriol, 1,25-dihydroxyvitamin D_3_ (1,25(OH)_2_D_3_), a bioactive form of vitamin D, and the role of MED28 in the progression of EMT in human colorectal cancer cells. Suppression of MED28 increased the expression of E-cadherin and reduced the expression of several mesenchymal and migration biomarkers and Wnt/*β*-catenin signaling molecules, whereas overexpression of MED28 enhanced the EMT features. Calcitriol suppressed the expression of MED28, and the effect of calcitriol mirrored that of MED28 silencing. Our data indicate that calcitriol attenuated MED28-mediated cell growth and EMT in human colorectal cancer cells, underlining the significance of MED28 in the progression of colorectal cancer and supporting the potential translational application of calcitriol.

## 1. Introduction

Biological features of malignant tumor cells include unchecked cell growth, epithelial-mesenchymal transition (EMT), invasion to neighboring tissues, and metastasis at distant sites [1]. During the development of invasive cancer, cells gradually lose epithelial features and acquire mesenchymal and invasive characteristics. E-cadherin is an epithelial junction protein responsible for the maintenance of the epithelial status. Three groups of transcription factors, Snail zinc finger family, zinc finger E-box-binding homeobox family proteins, and basic helix-loop-helix family can repress the expression of E-cadherin [2]. As in the case of colorectal cancer (CRC), cancer cells may display loss of polarity, epithelial markers such as E-cadherin, and upregulation of mesenchymal markers such as vimentin as well as metalloproteinases MMP2 and MMP9 [3]. Intermediate filament vimentin contributes to the change of cell shape, motility, and adhesion during the progression of EMT [4]. Loss of E-cadherin, together with dysregulation in the Wnt/*β*-catenin pathway, a common theme in colorectal cancer, is implicated in the progression of cancer presumably by promoting EMT, invasion, and metastasis [5].

Skin cells produce vitamin D_3_ (cholecalciferol), a natural form of vitamin D, through a series of reactions upon sun exposure. Dietary sources of vitamin D are limited to mainly fortified foods and fish oils. After two hydroxylation events at C-25 and C-1 of cholecalciferol in the liver and kidneys, respectively, our body can produce 1,25-dihydroxyvitamin D_3_ (1,25(OH)_2_D_3_), *i.e.*, calcitriol, the biologically active form of vitamin D. The genomic action of calcitriol is mediated through binding to a heterodimer, vitamin D receptor (VDR) and retinoid X receptor (RXR), two steroid receptor family members [6]. The complex then recognizes and binds to vitamin D response element (VDRE) of the target genes to activate or repress gene expression [7]. Calcitriol can be also involved in nongenomic, transcription-independent signaling in certain contexts [8]. In terms of physiological roles, in addition to the maintenance of calcium and bone homeostasis, the initially identified function, calcitriol also exhibits other important biological effects on normal cell differentiation, immune function, and cancer, among others. *In vitro* studies have shown anticancer effects of calcitriol, including antiproliferation, prodifferentiation, and apoptosis [7, 9]. Vitamin D deficiency may increase the risk of developing multiple types of malignancies [9]. Most epidemiological studies report an inverse tendency between 25-hydroxyvitamin D_3_ (25(OH)D_3_) and cancer, including colorectal cancer [9–11]. Therefore, maintenance of adequate vitamin D status appears to reduce the incidence and mortality of colorectal cancer [12].

Mediator subunit MED28 exhibits multiple cellular roles, including facilitating transcriptional activation [13] and interacting with signaling molecules such as Grb2 and Src family proteins [14–16]. Recently, we have reported that MED28 appears involved in Wnt/*β*-catenin signaling in human colorectal cancer cells [17] as well as EMT and migration in human breast cancer cells [18, 19]. However, whether MED28 is also involved in the EMT event in colorectal cancer is unclear at present.

The aim of this study was to investigate the role of MED28 in the progression of EMT and a potentially suppressive effect of calcitriol, the bioactive form of vitamin D, on MED28 and EMT in human colorectal cancer cells. Suppression of MED28 by RNA interference upregulated E-cadherin, an epithelial marker, but downregulated several mesenchymal biomarkers as well as Wnt/*β*-catenin signaling molecules, whereas overexpression of MED28 affected EMT in an opposite manner. Calcitriol suppressed the expression of MED28, and the effect of calcitriol mirrored that of MED28 silencing. Moreover, calcitriol could reverse the stimulatory effect of MED28 on EMT. Our data indicate that calcitriol attenuated MED28-mediated cell growth and EMT in colorectal cancer cells, reinforcing the implication of MED28 in the progression of colorectal cancer and supporting a promising, clinical application of calcitriol.

## 2. Materials and Methods

### 2.1. Cell Culture

The chemicals and cell culture supplies stated in the current study were purchased from MilliporeSigma (Burlington, MA) and Invitrogen (Carlsbad, CA), respectively, unless stated otherwise. Human colorectal cancer cell lines, HT29 and SW480 (American Type Culture Collection, Manassas, VA), were cultured in 10% fetal bovine serum-containing Dulbecco's modified Eagle's medium and Leibovitz's L-15 medium without CO_2_, respectively. For RNA interference experiments, cells were transfected with MED28-specific small interfering RNA pools (Thermo Fisher Scientific, Lafayette, CO) using Lipofectamine™ transfection RNAiMAX Reagent. For overexpression experiments, cells were transfected with Flag-MED28 cDNA (OriGene Technologies, Inc., Rockville, MD) using Lipofectamine™ 3000 Transfection Reagent.

### 2.2. Preparation of Total Cell Lysates and Western Blotting

After the addition of 100 nM of calcitriol (Cayman Chemical, Ann Arbor, Michigan) for 48 h or transfection for 24 h (overexpression) or 72 h (RNAi), HT29 or SW480 cells were washed with PBS briefly, and the preparation of total cell lysates was as stated before [20]. Cellular proteins of the whole cell lysates were separated and resolved by SDS–PAGE and electrophoretically transferred onto polyvinylidene fluoride membranes (Perkin Elmer Life Sciences, Inc., Waltham, MA). Western blotting was performed as previously described [20] with the following primary and secondary antibodies: MED28, *β*-catenin, and p-GSK3*β* (Ser9) antibodies from MilliporeSigma (Burlington, MA); fibronectin, E-cadherin, MMP2, and MMP9 antibodies from Abcam (Waltham, MA); anti-Flag-tag and anti-p21 from Proteintech Group, Inc. (Rosemont, IL); antibodies for vimentin, Snail, Slug, Twist, ZEB2, proliferating cell nuclear antigen (PCNA), *α*-tubulin, *β*-actin, vinculin, and GAPDH from Santa Cruz Biotechnology (Santa Cruz, CA); and anti-c-Myc, anticyclin D1, and goat antirabbit or antimouse-conjugated horseradish peroxidase secondary antibodies from GeneTex, Inc. (Irvine, CA).

### 2.3. RNA Extraction and Quantitative Real-Time Polymerase Chain Reaction (qRT-PCR)

Following the addition of 100 nM of calcitriol for 48 h, HT29 or SW480 cells were washed with PBS briefly, and the protocols of the RNA extraction and cDNA synthesis using Total RNA Mini Kit (NovelGene, Taipei, Taiwan) and iScript™ cDNA Synthesis Kit (Bio-Rad, Hercules, CA), respectively, were as described before [21, 22]. We employed the Bio-Rad products, including iQ™ SYBR® Green Supermix, CFX Connect™ Real-Time PCR Detection System, and CFX Manager™ 3.0 Software, for quantification, detection, and analysis, respectively. The following PCR primers were used: MED28 (forward, 5′-TTCGAACCGGTGTTGATCAG-3′; reverse, 5′-GCCAATGCCTCAGCTTTGTC-3′); GAPDH (forward, 5′-CGACCACTTTGTCAAGCTCA-3′; reverse, 5-AGGGGAGATTCAGTGTGGTG-3′).

### 2.4. Xenograft Mice

The animal study with the approved protocol (CJCU-104-001) issued by the Institutional Animal Care and Use Committee, Chang Jung Christian University (Tainan, Taiwan), was previously described [20]. Briefly, three-week-old male NOD/SCID mice were allowed for one week of acclimation after transferred from the National Cheng Kung University Animal Center (Tainan, Taiwan). These mice were subcutaneously implanted with human colorectal cancer HT29 cells (5 × 10^6^) to their flank regions, followed by random allocation into three groups, with four mice in each group. Two weeks later, each mouse was intraperitoneally administered with the, respectively, assigned dose, 0 *μ*g (vehicle), 0.5 *μ*g, or 1 *μ*g of calcitriol every other day. After 4 weeks, these mice were sacrificed, and their tissue sections and proteins were subjected to immunohistochemical or Western blotting analysis.

### 2.5. Immunohistochemical Analysis

Xenograft tumors were fixed in 4% paraformaldehyde, cut with a thickness of 5 *μ*m, and deparaffinized with xylene. Endogenous peroxidase was quenched with 3% H_2_O_2_ followed by dehydration with ethanol and antigen retrieval in a microwave for 5 min. After blocking with 5% bovine serum albumin solution for 1 h, tissue sections were incubated with anti-MED28 antibodies (1 : 50) for 2 h followed by secondary antibodies for 1 h and 3,3′-diaminobenzidine tetrahydrochloride. Immunostaining was observed under Nikon (Eclipse 50i) light microscope at 400× and photographed.

### 2.6. Statistical Analysis

Data are presented as means ± standard deviation which was analyzed by Student's *t* test or the analysis of variance (ANOVA) test followed by Tukey's post hoc comparison to analyze the differences between groups as appropriate. The results were considered significantly different at *p* < 0.05.

## 3. Results

### 3.1. *In Vivo* Effect of Calcitriol on the Expression of E-Cadherin, MED28, *β*-Catenin Signaling Molecules, and PCNA

**Figure 1:**
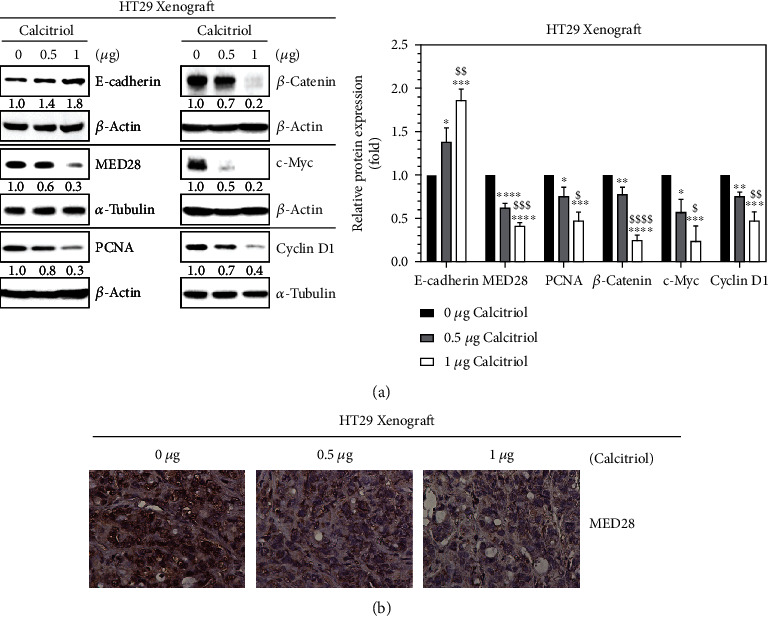
Calcitriol reduced the expression of MED28, PCNA, *β*-catenin, c-Myc, and cyclin D1 but upregulated the expression of E-cadherin in the subcutaneous HT29 xenografts. Four-week-old male NOD/SCID mice were subcutaneously implanted with human colorectal cancer HT29 cells to their flank regions, and these animals were then randomly allotted into three groups, vehicle control (0 *μ*g) or calcitriol (0.5 *μ*g or 1 *μ*g), with four mice in each group. Two weeks later, the animals were intraperitoneally administered with the assigned dose, 0 *μ*g (vehicle), 0.5 *μ*g, or 1 *μ*g of calcitriol every other day. The animals were sacrificed after 4 weeks. (a) Representative images and densitometric quantification for the relative protein expression of the xenografts. The expression levels of E-cadherin, MED28, PCNA, *β*-catenin, c-Myc, and cyclin D1 in the xenografts, along with loading controls, were detected by Western blotting. The ratios below the images (left panel) indicate the relative expression of the specific proteins with respect to those of 0 *μ*g after normalization with the expression of the corresponding loading controls, *β*-actin or *α*-tubulin. Densitometric data (right panel) are expressed as means ± standard deviation, *n* = 3; ^∗^*p* < 0.05, ^∗∗^*p* < 0.01, ^∗∗∗^*p* < 0.001, and ^∗∗∗∗^*p* < 0.0001 as compared with 0 *μ*g; ^$^*p* < 0.05, ^$$^*p* < 0.01, ^$$$^*p* < 0.001, and ^$$$$^*p* < 0.0001 as compared with 0.5 *μ*g. (b) Tissue sections of the xenografts were subjected to immunohistochemical analysis with anti-MED28 antibodies followed by secondary antibodies and diaminobenzidine staining. Brown-colored immunostaining was observed under Nikon (Eclipse 50i) light microscope at 400× and photographed.

Previously, we employed a subcutaneous HT29 xenograft NOD/SCID mouse model to examine the *in vivo* effect of calcitriol (bioactive vitamin D), and we found a dose-dependent tendency of calcitriol in reducing the tumor volumes and weights of the xenografts [20]. Loss of cadherin-mediated adhesion is implicated in the progression of cancer [5], and calcitriol upregulates the expression of E-cadherin in HT29 and SW480 human colorectal cancer cells [20]. In the current study, we further identified that calcitriol increased the expression of E-cadherin in the subcutaneous HT29 xenografts (Figure 1(a)). In an earlier study, we reported that MED28 appears to regulate Wnt/*β*-catenin signaling and cell growth in human colorectal cancer cells [17]. Therefore, we asked whether calcitriol may affect the expression of MED28, Wnt/*β*-catenin signaling molecules, and cell growth *in vivo*. As shown in Figure 1, both Western blot (Figure 1(a)) and immunohistochemical (Figure 1(b)) analyses revealed that calcitriol reduced the expression of MED28 in a dose-responsive manner in the HT29 xenografts. Moreover, calcitriol reduced the expression of PCNA, a proliferation marker, and Wnt/*β*-catenin signaling molecules, including *β*-catenin, c-Myc, and cyclin D1, in the HT29 xenografts (Figure 1(a)). These data indicated an *in vivo* effect of calcitriol on E-cadherin, MED28, Wnt/*β*-catenin signaling, and cell growth in human colorectal cancer.

### 3.2. Effect of Calcitriol on MED28, Epithelial and Mesenchymal Markers, and Wnt/*β*-Catenin Target Genes in Human Colorectal Cancer Cells

**Figure 2:**
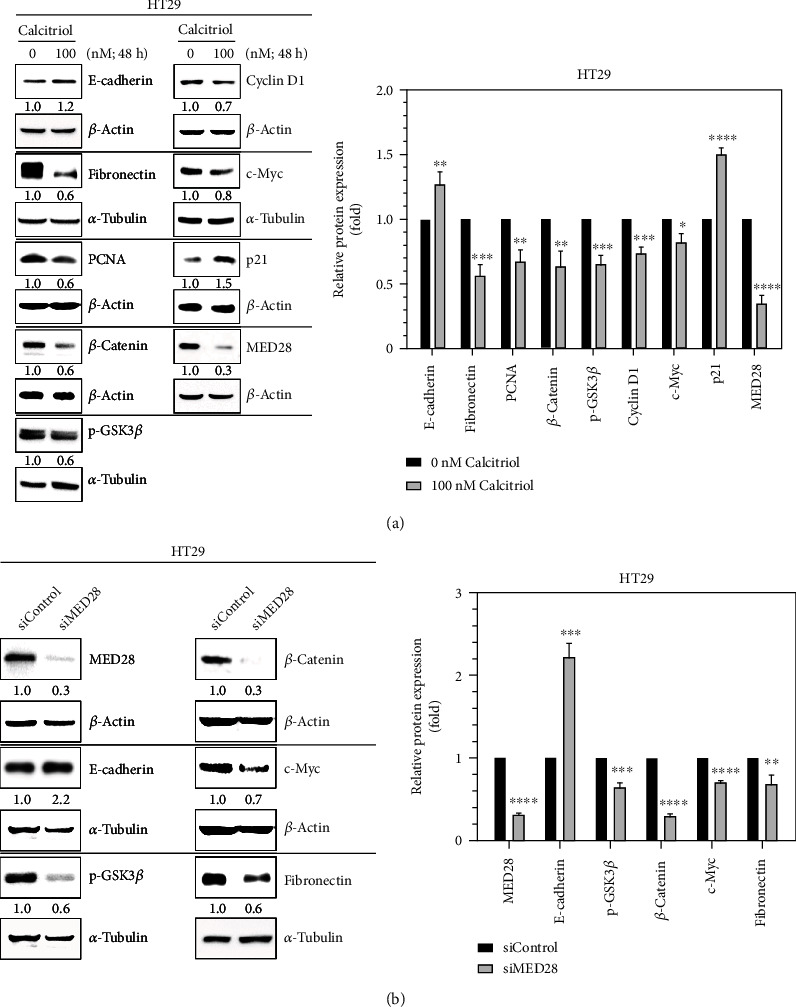
MED28 knockdown mimicked the effect of calcitriol on E-cadherin and Wnt/*β*-catenin signaling in HT29 human colorectal cancer cells. HT29 cells were treated with calcitriol (100 nM) for 48 h (a) or undergone MED28-specific siRNA (siMED28) for 72 h (b) with respective controls and subjected to Western blotting with the antibodies indicated. The ratios below the representative images (left panels) indicate the relative expression of the specific proteins with respect to those of 0 nM (a) or siControl (b) after normalization with the expression of the corresponding loading controls, *β*-actin or *α*-tubulin. Densitometric data (right panels) are expressed as means ± standard deviation, *n* = 3; ^∗^*p* < 0.05, ^∗∗^*p* < 0.01, ^∗∗∗^*p* < 0.001, ^∗∗∗∗^*p* < 0.0001 as compared with 0 nM (a) or siControl (b).

Wnt/*β*-catenin signaling is associated with EMT and migration during the development of malignancy [2]. Previously we found a link of MED28 with Wnt/*β*-catenin signaling in human colorectal cancer cells and a connection of MED28 and EMT in human breast cancer cells [17, 19]; we therefore asked whether calcitriol may also affect MED28 and EMT in colorectal cancer. In agreement with our previous findings [20], the addition of calcitriol at 100 nM for 48 h in HT29 cells reduced the expression of multiple proteins, including p-GSK3*β*, *β*-catenin, cyclin D1, and c-Myc, involved in Wnt/*β*-catenin signaling, and PCNA, but upregulated p21 (Figure 2(a)). Also, calcitriol suppressed the protein levels of fibronectin, a mesenchymal marker, and MED28, but upregulated the expression of E-cadherin (Figure 2(a)). Next, we employed RNAi to examine the role of MED28, and we found that suppression of MED28 mimicked the effect of calcitriol on E-cadherin, fibronectin, and Wnt/*β*-catenin signaling molecules in HT29 human colorectal cancer cells (Figure 2(b)). These data indicated that calcitriol may suppress the Wnt/*β*-catenin signaling pathway partially through MED28 in human colorectal cancer cells.

**Figure 3:**
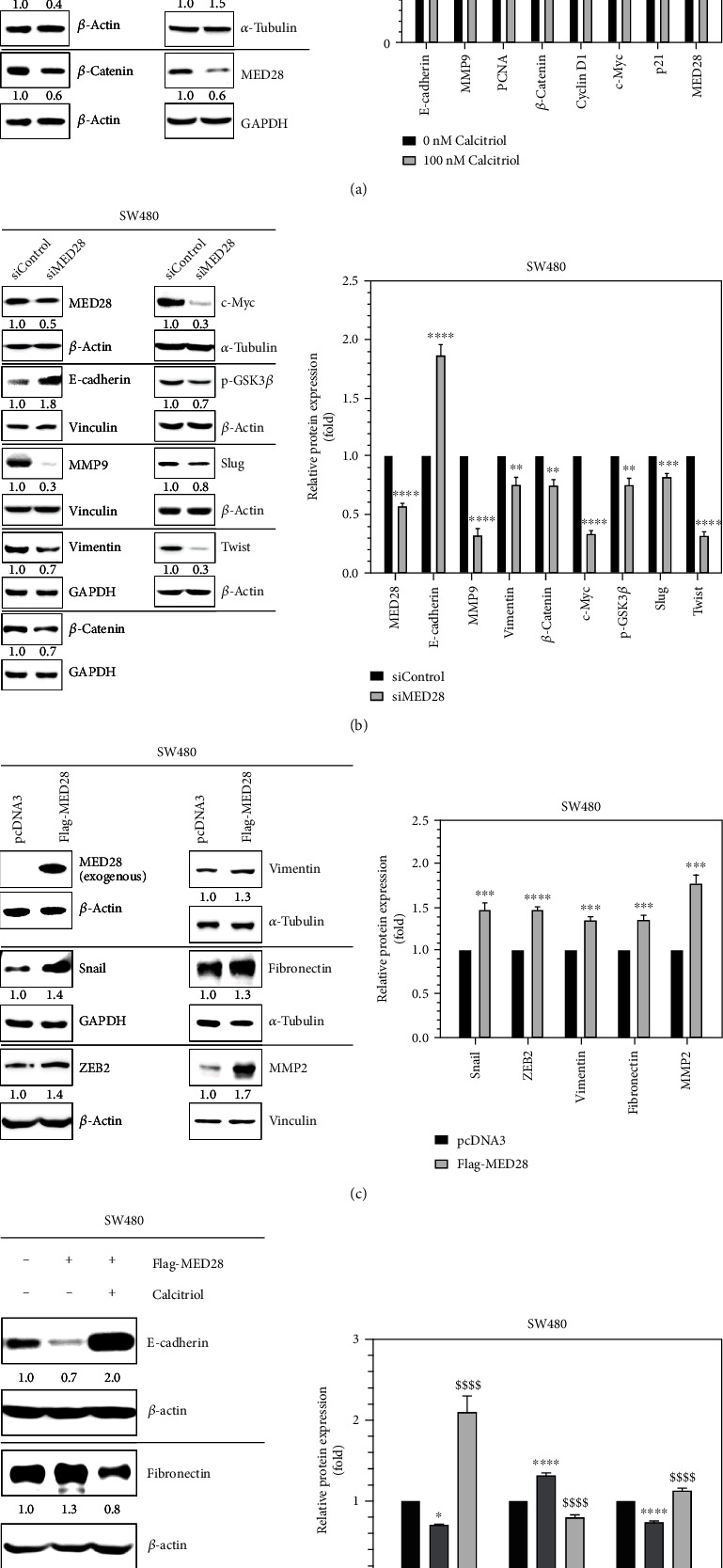
Calcitriol suppressed the upregulatory effect of MED28 on cell growth and epithelial-mesenchymal transition in SW480 human colorectal cancer cells. SW480 cells were treated with calcitriol (100 nM) for 48 h (a), undergone MED28-specific siRNA (siMED28) for 72 h (b), or MED28 overexpression plasmid (Flag-MED28) for 48 h (c), with respective controls, and subjected to Western blotting. The ratios below the representative images (left panels) indicate the relative expression of the specific proteins with respect to those of 0 nM (a), siControl (b), or pcDNA3 (c) after normalization with the expression of the corresponding loading controls, *β*-actin, *α*-tubulin, GAPDH, or vinculin. Densitometric data (right panels) are expressed as means ± standard deviation, *n* = 3; ^∗∗^*p* < 0.01, ^∗∗∗^*p* < 0.001, and ^∗∗∗∗^*p* < 0.0001 as compared with 0 nM (a), siControl (b), or pcDNA3 (c). (d) SW480 cells were transfected with MED28 overexpression plasmid (Flag-MED28), followed by the addition of calcitriol for 48 h, and then subjected to Western blotting. “–” indicates vehicle control (0 nM of calcitriol) or pcDNA3 transfection. The ratios below the representative images (left panel) indicate the relative expression of the specific proteins with respect to those of lane 1 (0 nM and pcDNA3) after normalization with the expression of the corresponding loading controls, *β*-actin, or GAPDH. Densitometric data (right panel) are expressed as means ± standard deviation, *n* = 3; ^∗^*p* < 0.05 and ^∗∗∗∗^*p* < 0.0001 as compared with lane 1 (0 nM and pcDNA3); ^$$$$^*p* < 0.0001 as compared with lane 2 (0 nM and Flag-MED28).

**Figure 4:**
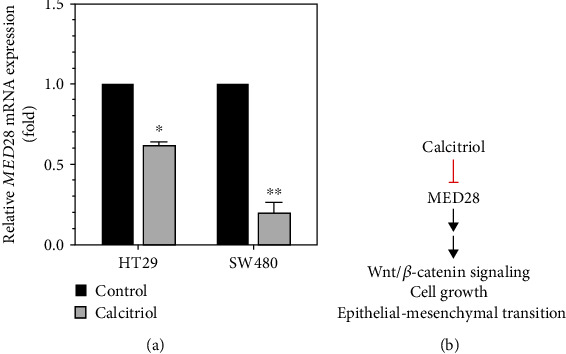
Calcitriol suppresses Wnt/*β*-catenin signaling, cell growth, and epithelial-mesenchymal transition, partially through downregulating MED28 expression, in human colorectal cancer cells. (a) SW480 and HT29 cells were treated with calcitriol (100 nM) for 48 h and subjected to RNA extraction and quantitative real-time polymerase chain reaction as described in Materials and Methods. Data are expressed as means ± standard deviation, *n* = 3; ^∗^*p* < 0.05 and ^∗∗^*p* < 0.01 as compared with the respective control (Control; 0 nM of calcitriol) after normalization with the expression of GAPDH. (b) MED28 upregulates Wnt/*β*-catenin signaling, cell growth, and epithelial-mesenchymal transition, which can be suppressed by calcitriol.

Next we investigated the effect of calcitriol on SW480, another human colorectal cancer cell line. The addition of calcitriol upregulated the expression of E-cadherin and p21 but suppressed the expression of PCNA, *β*-catenin, cyclin D1, c-Myc, and MMP9, a metalloproteinase involved in migration and invasion, and MED28 in SW480 cells (Figure 3(a)). Suppression of MED28 led to downregulation of MMP9, vimentin, and Slug and Twist, two transcription factors involved in EMT, in addition to Wnt/*β*-catenin signaling molecules such as p-GSK3*β*, *β*-catenin, and c-Myc, but upregulation of E-cadherin (Figure 3(b)). In contrast, overexpression of MED28 increased the expression of mesenchymal markers, including vimentin and fibronectin, and transcription factors Snail and ZEB2, but decreased the expression of E-cadherin and p21 (Figures 3(c) and 3(d)). However, calcitriol could reverse the effect of MED28, thereby upregulating E-cadherin and p21 and downregulating fibronectin (Figure 3(d)). The suppressive effect of calcitriol on MED28 appeared at the level of transcription because calcitriol reduced the mRNA expression of MED28 in both HT29 and SW480 cells (Figure 4(a)). Taken together, our data suggested that MED28 was involved in Wnt/*β*-catenin signaling and EMT, and calcitriol could inhibit the effect of MED28 on cell growth and EMT in colorectal cancer (Figure 4(b)).

## 4. Discussion

In the current study, we employed two human colorectal cancer cell lines, HT29 and SW480; these cells carry wild-type *CDH1* (E-cadherin) and *CTNNB1* (*β*-catenin), but mutant *APC* [23], and exhibit active Wnt/*β*-catenin signaling. We identified a role of MED28 in cell growth and epithelial-mesenchymal transition in these human colorectal cancer cells, where the effect of MED28 knockdown mimicked that of calcitriol, partially by upregulating E-cadherin as well as attenuating Wnt/*β*-catenin signaling and epithelial-mesenchymal transition (Figure 4(b)).

Cellular *β*-catenin may be present in multiple subcellular compartments including cell membrane, cytoplasm, and nucleus, and in normal cells, *β*-catenin is mainly found in cell–cell junctions associated with E-cadherin [24]. The distribution of cellular *β*-catenin is controlled by a multiprotein disruption complex, mainly consisting of axin, adenomatosis polyposis coli (APC), and glycogen synthase kinase 3*β* (GSK3*β*) [25]. In the absence of upstream stimulation, axin and APC hold *β*-catenin in place, and casein kinase 1 and GSK3*β* phosphorylate *β*-catenin at the designated amino acids residues, which ultimately lead to ubiquitination and proteasome-mediated degradation of cytosolic *β*-catenin. Upon activation, the interaction of Wnt and receptors rescues *β*-catenin from disruption, stabilizes *β*-catenin, and allows the entry of *β*-catenin into the nucleus [26]. *β*-catenin then forms a transcriptionally active complex with T cell factor (TCF) and lymphoid enhancer factor (LEF) and transactivates various downstream targets involved in cell growth such as cyclin D1 and c-Myc, as well as epithelial-mesenchymal transition (EMT) including fibronectin for mesenchymal adhesion and transcription factors Slug and Twist for transcriptional downregulation of E-cadherin. Therefore, E-cadherin and Wnt/*β*-catenin signaling are implicated in cancer development in the aspect of EMT, invasion, and metastasis [5].

The E-cadherin/*β*-catenin complex is involved in the stabilization of cell–cell contact for epithelial cells [27]. Damage of cadherin-mediated cell adhesion disrupts regular assembly of the epithelial structures, which could increase the likelihood of free cytosolic *β*-catenin translocating into the nucleus to transactivate the Wnt/*β*-catenin target genes [5], indicating a connection between loss of cadherin-mediated cell adhesion and activation of Wnt/*β*-catenin signaling. Ultimately EMT could ensue if accompanied by the induction of the mesenchymal markers. Calcitriol upregulated the expression of E-cadherin (Figures 1(a), 2(a), and 3(a)), which could presumably increase the likelihood of E-cadherin–*β*-catenin assembly at the adherens junction to maintain the integrity of the epithelial structure. In addition, MED28 downregulated the expression of E-cadherin, whereas calcitriol could not only reduce the expression of MED28 but also relieve the inhibitory restraint of MED28 for E-cadherin (Figures 1, 2, and 3). Both HT29 and SW480 cells express fibronectin without external induction. However, these cells exhibit differential protein expression of vimentin and E-cadherin. HT29 cells exhibit considerable, endogenous E-cadherin, but do not express vimentin by default. In contrast, SW480 cells express vimentin, but their E-cadherin expression is barely detectable unless subjected to ectopic induction such as calcitriol or MED28 silencing. Nevertheless, both MED28 knockdown and calcitriol addition could downregulate the expression of fibronectin and upregulate that of E-cadherin in either HT29 or SW480 cells (Figures 2 and 3), and their control over EMT appeared partially through regulating transcriptional repressors of E-cadherin (Figures 3(b) and 3(c)). In addition to EMT, calcitriol could also exert its effect on cell growth. Calcitriol reduced the expression of PCNA, a marker for cell proliferation, in HT29 xenografts as well as HT29 cells and SW480 cells (Figures 1(a), 2(a), and 3(a)). Furthermore, calcitriol or MED28 knockdown reduced the expression of Wnt/*β*-catenin signaling molecules, including c-Myc, cyclin D1, *β*-catenin, and p-GSK3*β*, but upregulated p21 (Figures 1(a), 2, and 3). Therefore, either MED28 silencing or calcitriol intervention could probably suppress cell proliferation and rescue the loss of epithelial polarity to reverse malignancy.

Calcitriol appears to exert its genomic and nongenomic effects to modulate the development of colorectal cancer through multiple avenues [8]. For example, calcitriol upregulates E-cadherin, which presumably increases the likelihood of *β*-catenin staying at adherens junctions. In the current study, we showed that calcitriol upregulated the expression of E-cadherin (Figures 1(a), 2(a), and 3(a)). Vaughan-Shaw et al. identified that calcitriol upregulates the expression of E-cadherin at the stages of both transcription and translation using *ex vivo* (patient-derived epithelial organoids) and *in vitro* (multiple CRC cell lines) approaches [28]. Xin et al. reported that calcitriol increases the binding between E-cadherin and *β*-catenin in SW480 cells using immunoprecipitation assay [29]. The formation of the calcitriol/VDR complex sequesters *β*-catenin from interacting with TCF/LEF1, which suppresses the transcriptional activation of the Wnt/*β*-catenin target genes [8]. Previously using the TOPFlash/FOPFlash reporter system, our laboratory has shown that both calcitriol addition and MED28 suppression downregulate the transcriptional activation of the Wnt/*β*-catenin signaling in SW480 cells [17, 20]. It is noteworthy that the genomic action of vitamin D exerts its effect on transcription through VDR, by interacting with other transcription factors and Mediator complex [7]. Therefore, through VDR-Mediator association, vitamin D could presumably regulate tumorigenesis. Interestingly, we found that calcitriol suppressed the mRNA expression of MED28 (Figure 4(a)), and MED28 is a Mediator subunit involved in the transcriptional activation of the RNA polymerase II-encoding genes [13]. Therefore, our data indicated that calcitriol could also exert its inhibitory effect on its downstream targets such as Wnt/*β*-catenin signaling molecules through suppressing the gene expression of MED28. Considering MED28 as a subunit of the Mediator complex and a link with Wnt/*β*-catenin signaling, it is intuitive to propose MED28 as a central player involved in the effect of calcitriol on the development of colorectal cancer.

In this study we identified a connection of MED28 with EMT and cell growth in human colorectal cancer cells such that MED28 regulates Wnt/*β*-catenin signaling and controls transcription factors involved in downregulating E-cadherin and upregulating mesenchymal markers such as MMP9 and fibronectin. The growth-promoting and EMT-upregulating modes of MED28 could be repressed by the addition of calcitriol (Figures 3(d) and 4(b)). Calcitriol may suppress the development of colorectal cancer through multiple pathways [8]. The reasons that we focused on Wnt/*β*-catenin signaling include a threefold explanation: (1) Wnt/*β*-catenin signaling, one of the major players in the progression of CRC, regulates cell growth and epithelial-mesenchymal transition in CRC. (2) MED28 regulates Wnt/*β*-catenin signaling and the expression of its downstream targets. (3) Calcitriol suppressed the expression of MED28 and Wnt/*β*-catenin signaling molecules. Therefore, we propose that one suppressive effect of calcitriol on CRC may work through MED28-mediated Wnt/*β*-catenin signaling. Calcitriol exhibits promising clinical application. For example, Yu et al. reported that calcitriol confers radiosensitivity in colorectal cancer, suggesting a synergistic effect between ionizing radiation and calcitriol [30]. Together, our data support that MED28 plays an important role in the development of colorectal cancer and indicate that calcitriol may be translationally applicable as an adjuvant in fighting this malignancy.

## Data Availability

The data that support the findings of this study are available from the corresponding author upon reasonable request.
